# Protonation Equilibrium in the Active Site of the Photoactive Yellow Protein

**DOI:** 10.3390/molecules26072025

**Published:** 2021-04-02

**Authors:** Pablo Campomanes, Stefano Vanni

**Affiliations:** Department of Biology, University of Fribourg, Chemin du Musée 10, 1700 Fribourg, Switzerland; stefano.vanni@unifr.ch

**Keywords:** photoactive yellow protein, low-barrier hydrogen bond, molecular dynamics, QM/MM, density functional theory

## Abstract

The role and existence of low-barrier hydrogen bonds (LBHBs) in enzymatic and protein activity has been largely debated. An interesting case is that of the photoactive yellow protein (PYP). In this protein, two short HBs adjacent to the chromophore, *p*-coumaric acid (pCA), have been identified by X-ray and neutron diffraction experiments. However, there is a lack of agreement on the chemical nature of these H-bond interactions. Additionally, no consensus has been reached on the presence of LBHBs in the active site of the protein, despite various experimental and theoretical studies having been carried out to investigate this issue. In this work, we perform a computational study that combines classical and density functional theory (DFT)-based quantum mechanical/molecular mechanical (QM/MM) simulations to shed light onto this controversy. Furthermore, we aim to deepen our understanding of the chemical nature and dynamics of the protons involved in the two short hydrogen bonds that, in the dark state of PYP, connect pCA with the two binding pocket residues (E46 and Y42). Our results support the existence of a strong LBHB between pCA and E46, with the H fully delocalized and shared between both the carboxylic oxygen of E46 and the phenolic oxygen of pCA. Additionally, our findings suggest that the pCA interaction with Y42 can be suitably described as a typical short ionic H-bond of moderate strength that is fully localized on the phenolic oxygen of Y42.

## 1. Introduction

Low-barrier hydrogen bonds (LBHBs) have been proposed to play an important role in enzymatic activity [1,2,3,4]. Their potential implications in the structure-based design of new drugs and enzyme inhibitors have also received attention and have been discussed in several studies [5,6,7]. LBHBs are structurally characterized by a hydrogen bond donor–acceptor distance of about 2.5 Å, shorter than that found in ordinary hydrogen bonds (HBs) (2.8–3.0 Å). However, a short donor–acceptor distance is not the only requisite to conclude that a particular HB is an LBHB: LBHBs also require proton sharing between the two electronegative heteroatoms (which must, in theory, present identical or very similar pKa values) instead of exclusive localization on one of them as in the case of the so-called short ionic HBs (SIHBs). Moreover, both a far-downfield ^1^H NMR (nuclear magnetic resonance) chemical shift (17–21 ppm) and a low deuterium fractionation factor (~0.3) are spectral features that have been found to further evidence the existence of LBHBs [2]. Therefore, not unexpectedly, neutron diffraction and NMR spectroscopy are techniques widely used to indicate, although not to fully verify, the presence of LBHBs in proteins. In addition, theoretical methods can also be used for a more complete physicochemical characterization of LBHBs. In an LBHB, the energy barrier between the two minimum structures (each of them corresponding to the classical configuration, in which the hydrogen is attached to one of the heteroatoms involved in the bond) is very low, and the proton can freely move along the reaction path joining the donor and the acceptor atoms. This leads to a situation where hydrogen bonding to both heteroatoms contains quasi-covalent features, in contrast with the important electrostatic contribution typically observed in SIHBs. Moreover, delocalization of the proton leads to a picture where it is located nearly at the same distance from the donor and the acceptor as the prototypical signature of LBHBs in neutron diffraction measurements.

**Figure 1:**
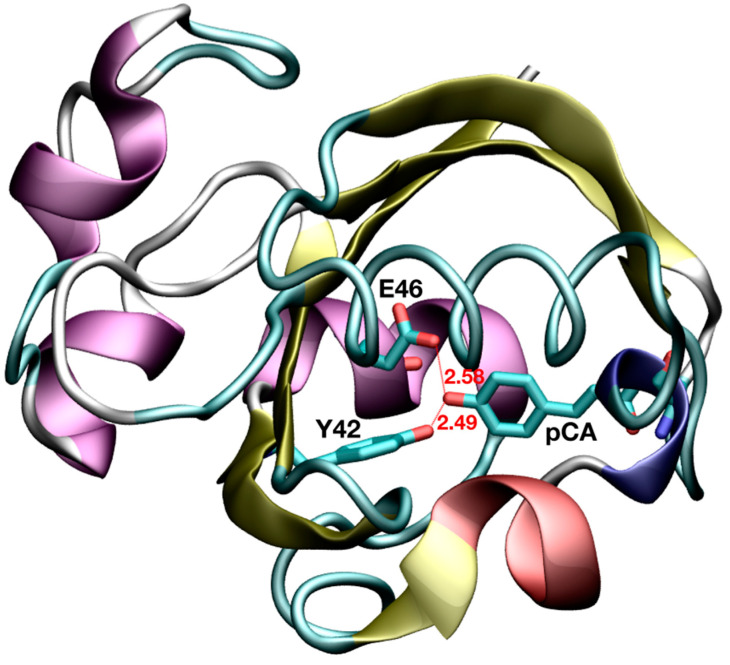
Short hydrogen bond interactions between active site residues and the photoactive yellow protein (PYP) chromophore. Distances are given in Å.

Thanks to the advancements in X-ray and neutron diffraction methodologies in the last few decades, short donor–acceptor distances have been revealed in a number of proteins [4]. In particular, both techniques have allowed the identification of two short HBs adjacent to the chromophore in the photoactive yellow protein (PYP) [8,9]. PYP is a light sensor that regulates the negative phototactic response in the halophilic photosynthetic bacterium *Halorhodospira halophila*, which belongs to the PER-ARNT-SIM (PAS) domain superfamily [10]. Its natural chromophore, *p*-coumaric acid (pCA), covalently attaches to C69 via a thioester bond and, upon light absorption, undergoes trans-cis isomerization of one of its double bonds. This double bond isomerization initiates a cascade of events which ends with protein unfolding into its signaling state [11,12]. The aforementioned short HBs are formed upon chromophore binding, in the dark state, between pCA and two residues in the binding pocket (E46 and Y42). In a high-resolution crystal structure of PYP [8], the O···O distances from the phenolic oxygen of pCA (O_pCA_) to the carboxylic oxygen of E46 (O_E46_) and from O_pCA_ to the phenolic oxygen of Y42 (O_Y42_) were found to be 2.58 and 2.49 Å, respectively (Figure 1). A subsequent study based on an ultra-high-resolution crystal structure (0.82 Å-resolution, code 1NWZ) of PYP has reported similar findings (O···O distances of 2.58 and 2.48 Å) [13]. Even at the high resolution of such X-ray structures, the location of the H atoms in these HBs has not been observed after model refinement and, based on the examination of spectroscopic and other structural data, it was concluded that none of these HBs could be considered as LBHBs [13].

However, a direct identification of the 87% H/D positions in PYP has been carried out in a more recent experimental study [14], where a high-resolution (1.5 Å) neutron crystallographic analysis was combined with high-resolution (1.25 Å) X-ray crystallography at room temperature (code 2ZOI). From the inspection of the measured interatomic distances (Table 1) in the binding pocket of the protein, the authors interpreted the pCA···E46 H-bond as an LBHB, with the deuterium atom delocalized in the central region between both oxygens. On the other hand, the pCA···Y42 interaction was assumed to be a typical SIHB, with the deuterium covalently bound to the phenolic oxygen of Y42. This lack of agreement in the nature of the above-mentioned H-bond interactions was further increased with the appearance of a very recent paper [15]. In this study, the authors calculated the residual electron density maps after the reexamination of the 1NWZ crystal structure. This strategy allowed them to conclude that the H atom located between pCA and E46 was, indeed, at only 0.92 Å from the carboxylic O atom of E46 and 1.67 Å from the phenolic O of pCA (O-H···O angle of 167 degrees). Therefore, as originally suggested, these new results indicate the absence of an LBHB between pCA and E46.

Several theoretical papers have also tried to mediate into this debate [16,17,18,19,20,21]. All of them support a scenario in which the pCA···E46 H-bond presents the typical features of an SIHB when PYP is in solution. Interestingly, one of them [19] also concluded that, in the solid (crystal) state, this hydrogen bond could be considered as an LBHB with the H shared between O_pCA_ and O_E46_. These theoretical studies have been either performed on (i) cluster models (without explicitly taking the effect of water solvation and protein environment into consideration), (ii) limited to static geometry optimizations (no thermal sampling into account), or (iii) based on semi-empirical hamiltonians to solve the electronic structure problem in quantum mechanical/molecular mechanical (QM/MM) approaches. However, three main factors are normally requested to obtain accurate and reliable results in theoretical studies of active site structural features and enzymatic reactivity: (i) an appropriate description of the active site interactions by means of density functional theory (DFT) or post-Hartree–Fock methods, (ii) an adequate treatment of water and protein environmental effects, and (iii) a proper thermal sampling of all statistically relevant configurations of the system under investigation. Therefore, in order to shed light on the above-described controversy, here, we investigated the dynamics of the protons involved in the two short HBs that, in the dark state of PYP, connect the chromophore with E46 and Y42 by means of DFT-based quantum mechanical/molecular mechanical (QM/MM) simulations. This approach allowed us to obtain a dynamical picture of the chromophore-protein interactions in solution, while appropriately taking into account thermal fluctuations. In this way, we investigated the combined effect of these two factors on the controversial short HBs found in the solved PYP structures.

## 2. Methods

### 2.1. System Setup

The model used as the starting configuration for the classical molecular dynamics (MD) simulations performed in this study was built based on the 1.5 Å-resolution neutron diffraction structure of PYP (PDB-ID: 2ZOI), which includes the 87% of the H/D atoms [14]. The side chain of R52 is deprotonated in the 2ZOI structure; however, it has been recently shown via NMR spectroscopy that R52 is, indeed, protonated in solution [22], as is expected from a solvent-exposed arginine at neutral pH. Therefore, it was treated as charged in our simulations. Conventional (pH = 7) protonation states for all the other titratable protein residues with unresolved H atoms were assigned using propKa [23,24], and further verified through inspection of their hydrogen bond pattern and nearby chemical environment. This structure was then immersed in a periodic box with a volume of ~60^3^ Å^3^, solvated with water molecules, and neutralized with Na^+^ counterions.

### 2.2. Classical Molecular Dynamics

The ff14SB parameterization of the all-atom AMBER force field [25] was used to model standard protein residues and counterions, whereas the TIP3P model [26] was employed for water molecules. For the chromophore, AM1-BCC atomic charges [27,28] were derived using antechamber [29] on a model system consisting of pCA and the cysteine covalently attached to it (C69). Electrostatic interactions were taken into account using the Particle Mesh Ewald (PME) algorithm [30] with a real space cutoff of 10 Å. The same cutoff was employed for the treatment of the van der Waals interactions. An integration time step of 1 fs was used. Room temperature (303.15 K) was achieved by coupling the system to a Langevin thermostat [31]. After minimization and careful thermalization of the PYP structure in water solution, a classical MD simulation was run in the isothermal-isobaric (NPT) ensemble for 100 ns to investigate the stability of the protein and the ability of the active site residues to sample different conformations. All the classical MD simulations were carried out using the NAMD (Nanoscale Molecular Dynamics) package [32]. The Visual Molecular Dynamics (VMD) software was used for visualization, analysis, and image generation [33].

### 2.3. QM/MM Molecular Dynamics

In order to choose a representative configuration to carry out the QM/MM MD study, we performed a clustering analysis [34] on the frames extracted from the above-mentioned classical MD simulation. Non-overlapping clusters were assigned using a local root mean square deviation (RMSD) (computed taking into account all protein residues within a 5 Å cutoff from the chromophore) as the measure of the distance between configurations. The central structure from the most populated cluster was then used to initiate the QM/MM MD simulation.

The QM/MM implementation employed in this study combines the use of the QM program QUICKSTEP [35] and the MM driver FIST, both forming part of the CP2K package [36]. In this code, the general QM/MM scheme is based on a real space multigrid technique to compute the electrostatic coupling between the QM and MM regions [37,38]. A quantum region (comprising the pCA chromophore and the side chains of the residues either involved in short HBs with pCA phenolic oxygen (Y42 and E46) or directly attached to it (C69)) was treated at the DFT level. The remaining part of the system, including water molecules and counterions, was modeled at the classical level using the AMBER force field. The valence of the terminal QM atoms was saturated by the addition of capping hydrogen atoms. A dual basis set, Gaussian and plane-wave (GPW) formalism, was employed to compute the interaction energy within the atoms belonging to the QM region. In particular, a double-ζ valence basis set augmented with a set of polarization functions (DZVP) [39] was used in order to obtain an accurate description of the wave function of the QM subsystem, while an auxiliary plane-wave basis set expanded up to a density cutoff of 320 Ry was utilized to converge the electron density in conjunction with Goedecker–Teter–Hutter (GTH) pseudopotentials [40,41] to describe the core electrons. Exchange and correlation energies were computed within the Generalized Gradient Approximation (GGA) using the BLYP functional [42,43]. The QM/MM MD simulations were performed with Born−Oppenheimer dynamics in the canonical (NVT) ensemble and using an integration time step of 0.5 fs. A self-consistent field (SCF) convergence threshold of 10^−6^ au was used to optimize the wave function at every step of the dynamics. Starting from the snapshot extracted from the forcefield-based dynamics, the system was equilibrated at the QM/MM level for about 2 ps. Then, the simulation was extended by 10 ps using stochastic velocity rescaling thermostats [44], independently for the QM and MM regions, to maintain the temperature of both at 303.15 K. [32].

### 2.4. Topological Analysis of the Electron Density

The topological features of the electron density for a representative configuration extracted from the QM/MM trajectory were characterized using Bader’s Quantum Theory of Atoms-in-Molecules (QTAIM) [45]. The topological analysis was carried out using the critic2 program [46] upon the addition of a core charge density to the original charge density, which contained only the contribution coming from the valence electrons because of the usage of pseudopotentials in our calculations. Kirzhnits’ approximation [47,48] was employed to estimate the value of the total electronic energy density at the bond critical points.

## 3. Results and Discussion

### 3.1. Binding Pocket and Protein Dynamics

**Figure 2:**
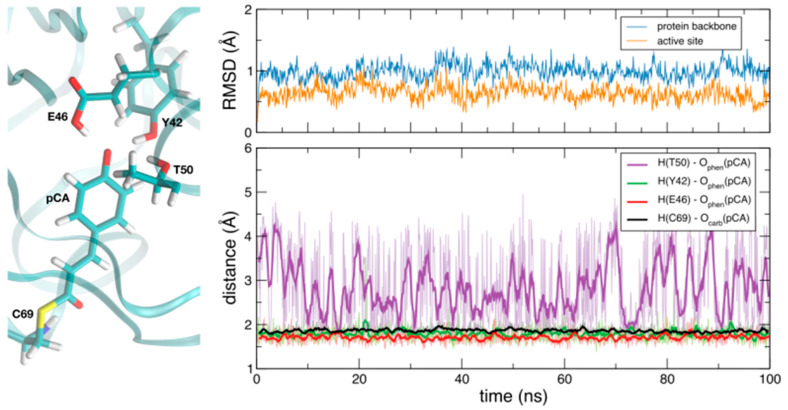
Left panel: PYP active site residues. Upper right panel: Time series of the root mean square deviation (RMSDs) for the protein backbone and the active site residues in the classical molecular dynamics (MD) simulations. 2ZOI was taken as reference structure to obtain the corresponding RMSD plots. Lower right panel: Time evolution of H···O distances that describe the interactions of the phenolic (O_phen_) and carbonyl (O_carb_) oxygen atoms of pCA with different active site residues. Values computed on equally spaced snapshots every 10 fs are displayed with pale colors. Colorful lines show their corresponding running averages (time window of 100 fs).

**Figure 3:**
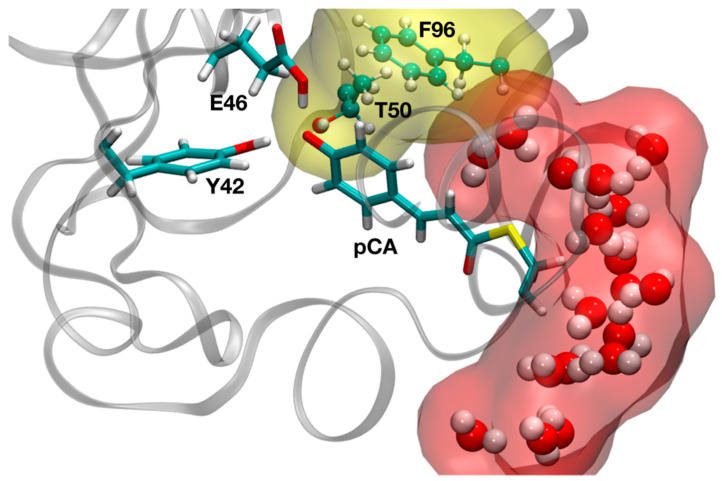
Representative snapshot from the classical MD simulation showing the orientation of T50 and F96 side chains, which impede the entrance of water molecules deeply into the region where the short hydrogen bonds (HBs) are located.

**Figure 4:**
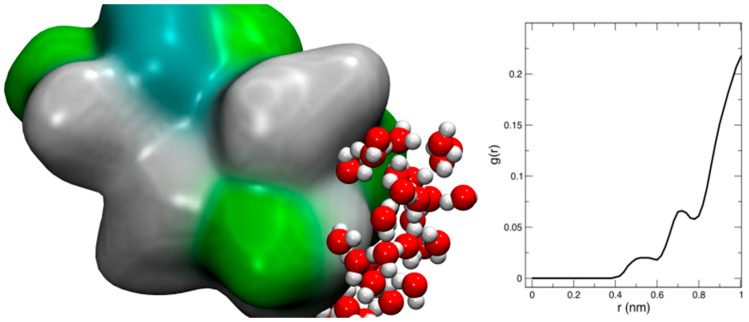
Left panel: Surface representation of the protein residues at the entrance of the binding cavity. The surface is colored according to the hydrophobicity and charge of the amino acids. Surface regions covering neutral hydrophobic and hydrophilic amino acids are depicted in grey and green, respectively, while those covering charged amino acids are colored in blue. The protein orientation is similar to that of Figure 3. Right panel: Radial distribution function of water oxygens around the phenolic oxygen of pCA.

As a first step in our study, we investigated the flexibility of the protein and the dynamics and possible hydration of the binding pocket by means of classical MD simulations. As clearly visible in the global RMSD plot obtained from a 100 ns-run of the chromophore-bound PYP in solution (Figure 2), the RMSD of the protein, with respect to the initial reference structure (2ZOI), fluctuates (after thermalization and subsequent equilibration) around an average value of 1.0 Å and no important protein conformational changes were observed in the timescale of the dynamics. This relatively low RMSD value is not unexpected, due to the high resolution of the neutron diffraction structure used to start the simulation. A similar behavior was displayed by a local RMSD defined by taking into account the residues that form the pocket where pCA binds (average RMSD of 0.6 Å). This reflects the stability of the dark-state PYP active site during the dynamics. As also shown in Figure 2, some important interactions in the binding pocket of the protein are maintained during the entire simulation time. That is the case of the HB between C69 and the carbonyl oxygen of pCA, which has been reported to play an important role in chromophore stabilization in the PYP ground state and in the photo-induced pCA isomerization [49], and the two short HBs between Y42 and E46 residues and the phenolic oxygen atom of pCA. T50, which is H-bonded to Y42 in the solved diffraction structures, shows a certain degree of flexibility during the dynamics and transiently binds to pCA (Figure 2). The pCA···Y42 H-bond distance (1.79 ± 0.07 Å) is slightly larger than that between pCA and E46 (1.71 ± 0.05 Å) on average. Interestingly, water molecules could not penetrate and solvate the region where these short HBs are located. This seems to be a consequence of the combined action of two residues: T50 and F96. The side chains of both residues (T50, with its OH group oriented pointing towards the interior of the cavity, and F96) are in the pathway that water molecules should follow to deeply invade the pCA binding pocket, and their presence creates a hydrophobic wall that blocks water diffusion into the neighborhood of the phenolic O of pCA (Figure 3 and Figure 4). Therefore, the vestibule to the pocket seems to be engineered to maintain a dry active site in the dark state. The absence of water molecules in the binding pocket during the dynamics is clearly manifested in the radial distribution function of water oxygens around the phenolic oxygen of pCA (Figure 4), which is a good measure of the probability for water molecules to solvate the region where the short H-bonds are located. In this dry environment, strong H-bond contacts could play an important role in order to stabilize the charge in the phenolic oxygen atom of the chromophore.

### 3.2. Proton Dynamics in the Active Site

The absence of important reorganizations in the interaction pattern of the active site residues during our classical MD simulations, confirmed by the RSMD plots displayed in Figure 2, facilitates the selection of a representative configuration to initiate the subsequent QM/MM MD simulation. Here, we used a local RMSD-based cluster analysis (see Methods for details) on the snapshots extracted from the classical simulation to pick an initial frame. As expected from the low variability shown in the RMSD, only one main cluster was obtained from this analysis. The central structure of this cluster was then employed as the starting configuration to investigate the nature of the short HBs found in the binding pocket of the pCA-bound protein by means of QM/MM dynamics.

To this end, we treated the protein chromophore and the side chains of Y42, E46, and C69 at the DFT level, while a classical forcefield was employed to model the environmental effects caused by other protein residues and water molecules (see Methods section for more details). Thermal sampling allowed us to explore the dynamics of the intermolecular HBs involving pCA and several active site residues. Remarkably, we observed proton hopping events taking place between the carboxylic O of E46 and the phenolic O of pCA in the sub-picosecond timescale, the distance between both oxygens being of about 2.5 Å on average. On the other hand, although the pCA···Y42 H-bond pattern was maintained during the 10 ps-long QM/MM simulation, we did not observe a similar proton transfer phenomenon in this case, with the hydrogen fully localized on the phenolic O of Y42 and at a distance of 1.00 ± 0.04 Å from it. Of interest, the pCA···C69 H-bond interaction was also preserved during the entire course of the simulation.

**Figure 5:**
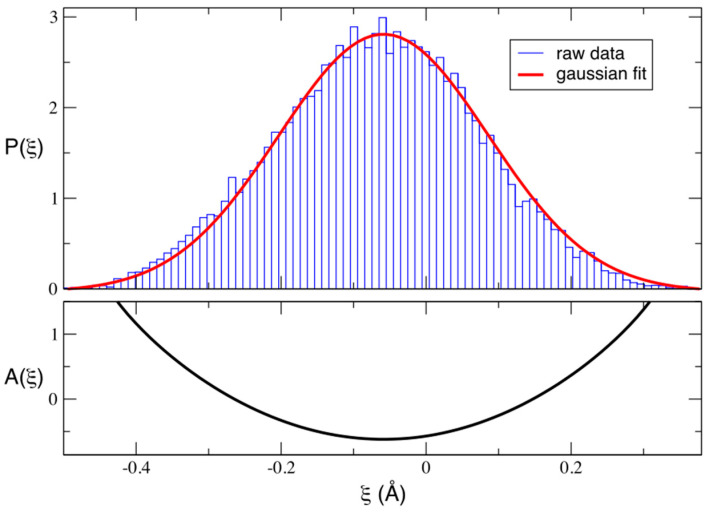
Upper panel: Normalized probability distribution, P(ξ) computed using equidistant frames extracted from the quantum mechanical/molecular mechanical (QM/MM) trajectory. The raw distribution is shown as a histogram, built using a bin width of 0.01 Å. A gaussian fit to the histogram is also displayed. Lower panel: Potential of mean force, A(ξ) in kcal mol−1, estimated by Boltzmann inverting the gaussian fit: $A\left(\xi \right)=-K_{B}TlnP\left(\xi \right)$.

We then defined the reaction coordinate for proton hopping, ξ, as the difference between the two interatomic distances involved in the process (i.e., ξ = OE46···H - H···OpCA) and estimated the potential of mean force (PMF) along this reaction coordinate directly from its corresponding probability distribution, P(ξ). As shown in Figure 5, P(ξ) is unimodal and can be well approximated by a gaussian distribution with a mean value of −0.06 Å and a standard deviation of 0.14 Å. According to our definition, positive/negative values of ξ imply that the H being transferred is closer to OpCA/OE46. Therefore, a value of ξ=−0.06 Å is indicative of a configuration in which the H atom is almost equidistant from both oxygens, although slightly closer to OE46. As a consequence, the corresponding PMF (estimated Boltzmann inverting P(ξ)) presents a single-well. This situation would agree with a double potential energy well with a low barrier for the proton transfer between the donor and acceptor atoms, which could be easily overcome at room temperature and sampled in QM/MM simulations, as all thermal and entropic contributions are intrinsically taken into account during the dynamics.

### 3.3. QTAIM Description of the Short HBs with E46/Y42

The delocalization of the H in the central region between both O_E46_ and O_pCA_ and at a slightly shorter distance from O_E46_ qualitatively agrees with the scenario depicted by the resolved neutron diffraction structure and, as a consequence, the interpretation of the pCA···E46 interaction as an LBHB [14]. On the other hand, the localization of the other H atom on O_Y42_ is also consistent with the assumption of pCA···Y42 as a typical SIHB, as concluded by the same authors [14]. However, a rigorous description of the chemical nature of these interactions requires complementing the results of our QM/MM simulations with a general theory of bonding. Therefore, to unravel the chemical nature and the degree of covalency of the short HBs located in the PYP active site, we performed a QTAIM topological analysis of the electron density, ρ, for a representative configuration extracted from the dynamics.

QTAIM has been successfully applied in numerous studies to characterize chemical bonding and interatomic interactions. According to the AIM theory, the properties at a bond critical point (BCP), first-order saddle-point in the electron density, and along the path connecting two nuclei can be employed to fully determine the interaction between them. In particular, the value of ρ at the BCP, $\rho_{BCP}$, serves as a good measure of the strength of the interaction, whereas the sign of $\nabla^{2}\rho$ at the BCP, $\nabla^{2}\rho_{BCP}$, allows, in general, to distinguish covalent interactions ($\nabla^{2}\rho_{BCP}<0$) from close-shell contacts ($\nabla^{2}\rho_{BCP}>0$), such as ionic, van der Waals, or ordinary H-bond interactions [45]. Moreover, as bond formation requires a molecular energy gain, it has been suggested [50,51] that the energetic characteristics at the BCPs must also be analyzed to completely describe the strength and degree of covalency of the interactions. In particular, it has been proposed [52] that the strength of HB interactions can be fully characterized by the sign of $\nabla^{2}\rho_{BCP}$ together with that of the total electronic energy density, $H_{BCP}$. Accordingly, weak HBs (interaction energies < 12 kcal/mol) should present both $\nabla^{2}\rho_{BCP}$ and $H_{BCP}>0$, moderate HBs (interaction energies 12–24 kcal/mol) could be described by $\nabla^{2}\rho_{BCP}>0$ and $H_{BCP}<0$, and strong HBs, including resonance-assisted HBs (RAHBs) and LBHBs, (interaction energies > 24 kcal/mol) should have both $\nabla^{2}\rho_{BCP}$ and $H_{BCP}<0$.

**Figure 6:**
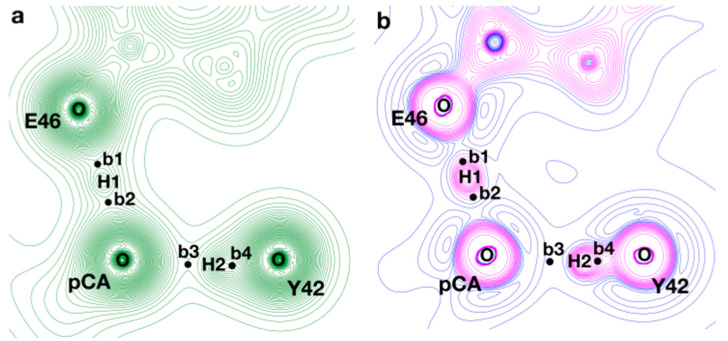
ρ (**a**) and $\nabla^{2}\rho$ (**b**) contour maps in the plane that contains the two short HBs between pCA and E46/Y42. The structure examined corresponds to a frame extracted from the QM/MM trajectory, which is representative of the minimum found in the potential of mean force (PMF). The black circles (**b1**–**b4**) represent the location of the bond critical points found between the atoms involved in the HBs. In the case of $\nabla^{2}\rho$, positive and negative contour lines are displayed in blue and magenta, respectively.

Within this framework, we performed a topological analysis of ρ for a structure representative of the minimum found in the PMF (ξ=−0.06 Å). In this structure, we examined the properties at the BCPs found along the short HBs between pCA and E46/Y42 to characterize these interactions and further validate the results of our simulations. As shown in Figure 6, the topology of ρ presents four BCPs (b1–b4) along the path connecting the proton-donating and proton-acceptor heteroatoms. The values computed for $\rho_{BCP}$, $\nabla^{2}\rho_{BCP}$, and $H_{BCP}$ at those critical points are displayed in Table 2. It is clear from Figure 6 that there is an important ρ accumulation along the path connecting OY42 and H2, while a depletion of electron density can be observed in the direction parallel to the bond path between H2 and OpCA. High values of $\rho_{BCP}$ and $\left|\nabla^{2}\rho_{BCP}\right|$ at b4, with both $\nabla^{2}\rho_{BCP}$ and $H_{BCP}<0$, confirm the existence of a covalent contact between H2 and OY42; in addition, a low value of $\rho_{BCP}$ (with $\nabla^{2}\rho_{BCP}>0$ and $H_{BCP}<0$) is found at b3; taken all together, these results allow us to describe the OY42···H···OpCA interaction as a short H-bond of a moderate strength with a close-shell contact between H2 and OpCA, which are typical attributes of SIHBs. On the other hand, intermediate values of $\rho_{BCP}$ at b1 and b2 on top of negative values of both $\nabla^{2}\rho_{BCP}$ and $H_{BCP}$ can be attributed to a scenario where the contacts of H1 with both oxygens present an important quasi-covalent contribution; therefore, the O_E46_···H···O_pCA_ interaction presents characteristics of a strong H-bond with a fingerprint that contains the typical features of an LBHB.

## 4. Conclusions

Here, we performed a computational study that combines classical and DFT-based QM/MM molecular dynamics simulations to shed light on the current controversy about the chemical nature and dynamics of the protons involved in the two short hydrogen bonds that, in the dark state of PYP, connect the protein chromophore with two binding pocket residues (E46 and Y42). This approach allowed us to obtain a picture of the chromophore–protein interactions in the solution, while appropriately taking into account thermal fluctuations and entropic effects on the dynamics of the system.

The analysis of our simulations reveals that these two HBs present distinct characteristics: O_Y42_···H···O_pCA_ can be described as a short ionic H-bond of moderate strength, in which the hydrogen is fully localized on the O atom of Y42 and covalently attached to it. Alternatively, O_E46_···H···O_pCA_ presents a fingerprint that contains the typical features of a strong LBHB. It has a low barrier for proton transfer between the donor and acceptor atoms, which can be easily overcome at room temperature, therefore, leading to complete delocalization of the H in the central region between both oxygens (at a slightly shorter distance from O_E46_). Furthermore, the contacts of this hydrogen with both oxygens present an important quasi-covalent contribution. This scenario qualitatively agrees with that depicted by the resolved neutron diffraction structure of PYP [14], supporting the existence of an LBHB in the PYP active site between the chromophore and E46. This LBHB could play an important role in order to stabilize the charge in the phenolic oxygen atom of the chromophore in the dry environment found in the protein binding pocket.

## Figures and Tables

**Table 1:** Distances, in Å, measured between the atoms involved in the short hydrogen bonds (HBs) with the chromophore (*p*-coumaric acid: pCA) in PYP active site.

pCA···Y42			pCA···E46		
d(O**_pCA_**-O**_Y42_**)	d(O**_pCA_**-H)	d(O**_Y42_**-H)	d(O**_pCA_**-O**_E46_**)	d(O**_pCA_**-H)	d(O**_E46_**-H)
2.52	1.65	0.96	2.56	1.37	1.21

**Table 2:** Electron density ($\rho_{BCP}$), laplacian ($\nabla^{2}\rho_{BCP}$), and total electronic energy density ($H_{BCP}$) calculated at the bond critical points (BCPs) found along the short HBs between pCA and E46/Y42. The locations of the BCPs (**b1**–**b4**) are shown in Figure 5. The distances (in Å) between every BCP and the atoms (O and H) that it connects are also given.

BCP	$\rho_{BCP}\;(a.u.)$	$\nabla^{2}\rho_{BCP}\;(a.u.)$	$H_{BCP}\;(a.u.)$	$d_{BCP-O}$	$d_{BCP-H}$
**b1** (OE46···H1)	1.70 × 10^−1^	−2.83 × 10^−1^	−1.74 × 10^−1^	0.91	0.31
**b2** (OpCA···H1)	1.44 × 10^−1^	−1.06 × 10^−1^	−1.23 × 10^−1^	0.94	0.34
**b3** (OpCA···H2)	6.71 × 10^−2^	1.51 × 10^−1^	−1.92 × 10^−2^	1.05	0.49
**b4** (OY42···H2)	3.42 × 10^−1^	−2.72	−7.08 × 10^−1^	0.77	0.21

## Data Availability

Not applicable.
